# Mistakes and Failures in Crown and Bridge-work

**Published:** 1902-04-15

**Authors:** F. E. Logan

**Affiliations:** Detroit, Mich.


THE
DENTAL REGISTER.

Vol. LVI. April 15, 1902. No. 4.

### MISTAKES AND FAILURES IN CROWN AND BRIDGE-WORK.

BY F. E. LOGAN, D.D.S., DETROIT, MICH.

Read before the Washtenaw Dental Society, March 1[0], 1902.

The subject of this paper will undoubtedly appeal to everyone here to-night, for we have all made mistakes and some of us have had failures in this particular branch of dentistry.

A great deal has been said and written lately that has been adverse to bridge-work, but why should we allow this adverse criticism to retard its progress and development? If bridge-work is properly constructed on suitable anchorages or abutments, it is the most useful and comfortable of all artificial substitutes, and fills a place in dentistry which can never be taken by anything else. If the work is practically and skillfully executed, no other method of procedure seems to offer so great an opportunity for the serviceable, and, I may say, permanent reproduction of the normal condition. But why so many failures? I shall endeavor to point out a few of the reasons.

A great many attempt the construction of bridge-work whose limits are rubber plates. Then again a large number of practitioners, already in the profession when modern bridge-work was introduced, have attempted this work having had very little or no instruction, and the natural result has been failure, because they did not possess a thorough knowledge and comprehension of the fundamental principles which underlie the adaptation of this complicated work. Others, who perhaps for the lack of judgment or love of the almighty dollar, have crowned or bridged everything that the fancy or the financial ability of their patients made possible. Consequently bridges are often misused by being inserted where a good plate would have been more satisfactory and serviceable to the patient.

Bridge-work has failed when it has been placed on weak or unhealthy roots, or where the tissues surrounding the roots are diseased. It stands to reason that if the abutments are not sound and strong, and the surrounding tissues not healthy, no matter how well a bridge has been constructed from the mechanical standpoint, it is sure to become a failure.

How often we see complete failure in this work on account of the faulty preparation of the tooth that is to serve as the abutment. If a dentist has not the time and patience to spend in properly preparing the tooth he had better pass bridge-work by and insert plates. It is absolutely essential that the abutment crown should fit perfectly at the gingival margin, otherwise the work can not be expected to be permanent. The tooth must be so shaped that when the enamel has been removed its greatest circumference is at the gum line. To procure this desired shape takes considerable skill, more especially in the molar region if the adjacent tooth is in place, for the disto-lingual angle is very difficult to prepare on account of the limited space. However, diamond disks, enamel cleavers and plenty of patience will soon reduce this bulge of tooth substance.

A root prepared in this manner is almost impervious to any external agents entering; but if it is done in a haphazard way, the crown fails to fit closely at the neck and as a result the cement dissolves, decomposing food and other conditions that favor decay, accumulate in the pocket previously filled by the cement and very soon the entire tooth is destroyed, sometimes without any warning to the patient. The preparation is one of the most important factors in successful bridge-work and nothing short of perfection will give us the results we seek to obtain.

Other failures have resulted from attaching too large a bridge to too few anchorages. I saw a good illustration of this in a patient's mouth where a dentist had inserted an all-gold bridge extending from a lateral incisor to a second molar. If that is not stretching bridge-work, what is? As a result of lateral force exerted in mastication, the abutments were forced out of place and the contour and symmetry of the face was completely spoiled.

Experience has taught us that the most successful bridges have been small ones which are attached to strong, healthy roots or teeth. Bridges of ten, twelve or fourteen teeth are seldom or ever admissable under any circumstance, unless the abutments are placed so as to equalize the pressure caused by the force of mastication.

A bridge is a rigid structure and when it is fixed to several supports, it can only assume the movement of one. The larger or more extensive the bridge, the more complicated become the forces that act upon it. Nature is generous in this direction, but there are cases when it sees fit to relieve itself of burdensome intrusions. There are certain prescribed limits wherein we are allowed to operate, and as soon as we understand and comprehend the principles and laws that should govern us within these limits and the failures and disasters that await us without, we shall have made some progress in the development of this most interesting work.

One of the gravest mistakes made to-day by the great majority of practitioners, lies in the use of bands and jacket crowns for anchorages in bridge-work; in ninety-nine percent of the cases they are an abomination and can not be too strongly condemned, more especially when the tooth tissue is lacking in lime salts and inclined to be weak. I care not how thoroughly the band or jacket has been adapted, the tooth substance is bound to disintegrate in a comparatively short length of time and we have nothing to show but a failure for a piece of work that was supposed to be permanent.

Besides the majority of the anterior teeth are so shaped that it is practically impossible to band them perfectly at the gingival margin without grinding the tooth so as to utterly ruin its appearance, for a tooth properly prepared for any crown or band must have its greatest circumference at the gum line.

Where jacket crowns or open-faced crowns are employed there occurs in nearly every instance a complete obliteration of the inter-proximal space; the gold comes in direct contact with the surface of the adjoining tooth and forms an admirable lodging place for debris, and in a few years you have a cavity in the adjoining tooth, a condition which would not likely have occurred if a Richmond crown had been employed.

Another pernicious practice is that of applying all-gold centrals and laterals. Is there anything more disgusting or inharmonious so far as esthetic appearances are concerned, than to see a patient with a flaring gold crown in the front of the mouth? Occasionally we encounter cases where a gold crown in this location is permissable. For instance in a man's mouth where the teeth have been worn down by mechanical abrasion, for in the majority of such cases the presence of the gold is hidden by the moustache; but in a woman's mouth, such procedure is entirely inexcusable, if not *criminal.* A few months ago a lady came into my office and requested that I apply an all-gold crown to her upper cuspid tooth. I suggested inserting a porcelain crown and explained to her how it would be more in harmony with the natural teeth in nearly every respect, and if the shade were perfect, it would be impossible to detect its presence. However, she would not listen to anything but a gold crown. I asked her why she preferred a gold crown and she could give no satisfactory reason any more than that she wanted it and was going to have one inserted. Getting somewhat out of patience with her, I remarked that she "reminded me of the natives in South Africa." She immediately asked, "in what respect." "Well," I answered, "they being unable to procure gold crowns, blacken all their teeth so they will not look like Europeans and Americans, who they claimed looked like dogs on account of their white teeth," For some reason I haven't seen her since.

The beauty of all artificial work lies in the ability of the artist to conceal his art, and nowhere is this more applicable than in crown and bridge-work. Do you think that bands, jacket crowns and gold crowns in the front of a patient's mouth assist in disguising it? I

claim they are very unsightly and decidedly inartistic. Suffice to say, that if dentistry is to become universally acknowledged as a profession, embracing a field of dignified and scientific pursuit; and if crown and bridge-work is ever to be accorded that recognition of an art to which scope its possibilities entitle it, the practice of placing bands and all-gold crowns on any of the six anterior teeth or within the range of vision, violates every principle of art, and must be degrading, and can not be too vigorously condemned, for I care not how well the work may have been performed from the mechanical standpoint, it is an offense to art and refinement.

There are practically but two permanent modes of anchorage for bridge-work—Richmond crowns for the six anterior teeth, and all-gold crowns for the posterior. By using these two modes of anchorages better and more permanent results will be obtained than if other and less secure methods are employed.

A properly constructed Richmond crown means a healthy well-filled root which should be cut off even with the gingival margin and on the labial surface slightly above it so that when the crown is completed no metal will be displayed to the vision. All enamel should be thoroughly removed and the end of the root reduced to the inverted cone shape—a neat, perfect-fitting 22k. gold band should be allowed to extend up under the gum line for about 1-32 of an inch. It is not necessary to force this band up until you squeeze the tears out of the patient's lachrymal gland, for if you do, your crown is worse than useless and it will very soon stir up a marked inflammation of the peridental membrane and surrounding tissues; the root becoming loose and painful. After the band is fitted, a thirty guage pure platinum cap plate is soldered to it and you have a cap which protects the root thoroughly. The root canal is then reamed out and a thirteen to sixteen guage irridio-platinum post is inserted after the cap has been put in place, both are then removed and united by soldering. After being replaced on the root and a porcelain facing ground to fit and properly backed with pure gold and platinum. The cutting edge being substantially protected, the facing is united with sticky wax to the cap, both are removed intact, invested and soldered. You now have one of the most perfect abutments for the anterior teeth that could possibly be constructed. It is artistic, strong and cleanly. There is absolutely no possibility of solution of cement and subsequent loosening of the piece as is liable where a band or jacket crown is attached.

*Anchorage in Fillings.* I have seen several pieces of bridge-work where the operator aimed to make a permanent piece of work by anchoring in adjoining teeth by means of fillings, either gold or amalgam, and I can not say that they were in first-class condition, nor is it surprising, considering that the principle is entirely at variance with the results desired. It is impossible to suppose that an occluso-proximal cavity of fairly good proportions can support the strain of mastication that is exerted on two, or even one, additional tooth, when experience teaches us that fillings in similar positions and of like dimensions frequently break down by force exerted on them ; for in the molar region, according to Dr. Black there is a strain of 70 to 250 pounds per individual tooth. Then furthermore, by virtue of the elasticity of the process and the joint peculiar to the teeth, there is always a certain amount of movement to overcome, and the security this mode of attachment offers, is as a rule far from sufficient; either the filling becomes loosened or the bar becomes detached. None of us could think of filling two opposing approximal cavities in adjoining teeth, with one filling and expect a successful operation. Yet the principle is identical. This class of work is not practical and seldom permissible; it has been successful only where the occlusion has been very slight or wanting.

A great amount of trouble has arisen from crowning and bridging teeth containing live pulps. The feasibility of sacrificing the pulps in teeth which are subsequently to be crowned is of the utmost importance, and should be carefully considered by every operator, for when the pulp has been left alive, we very often have subsequent troublesome symptoms which too frequently terminate in the death of the pulp, and this will surely be closely followed by disagreeable pathological conditions such as peridental inflammation and alveolar abscess.

Very often deleterious influences arise from the irritating action of the cement or acid used in mounting the abutments, but this can be almost entirely overcome if the rubber-dam is applied and the tooth thoroughly cauterized with silver nitrate before the crown is mounted, which will coagulate the albumen and prevent the action of thermal changes and the irritating cement influences.

Another serious trouble often occurs from excessive irritation following the preparation of the root before it is ready to receive the crown, such as filling, cutting and grinding, which are very often followed by marked congestion of the pulp, and unless it is soon relieved, results in the death of the same, and troubles commence.

All things considered, I think the most scientific treatment and by far the safest procedure in such cases is to devitalize and fill the canals thoroughly. However, if the preparation can be accomplished with little discomfort to the patient, which is frequently the case, by all means preserve the pulp's vitality.

It has been conceded by some of the best authorities in dentistry that the embryological if not the physiological function of the pulp discontinues with the complete development of the teeth, which is usually about the eighteenth or twentieth year, so that after the maturity of the teeth, the pulp is not absolutely necessary for its vitality or stability accordingly nothing is lost and a great deal is to be gained by destroying it when we have any doubts about its vitality, thus saving ourselves a world of trouble and our patients a deal of suffering at some future date.

This mode of treatment would not be advisable in the mouths of patients under eighteen years of age, for it is questionable if all the teeth are fully developed at that period. However, very little crown or bridge-work is required at that age. Nor is it necessary to destroy all pulps where the patient has reached the age of forty-five, because of the general atrophy of the pulp and the general deposit of dentin materially lessens the sensitiveness of the tooth, so that the necessary mechanical preparation can be accomplished with little or no discomfort to the patient. In such conditions we run very little chance of any future complications.

Faulty articulation will oftentimes play havoc with what might have otherwise been a perfect piece of work. The occlusion with the teeth in the opposing jaw has everything to do with the success of bridge-work, and nothing short of perfection in this particular will answer.

Bridges of sufficient solidity and with abutments equally firm are often capable of resisting any direct force or pressure caused by mal-occlusion, but may be torn to pieces by indirect impacts. The most disastrous of all these forces is the lateral, whereby the jaw is moved forward or sidewise against the bridge as a lever. In such cases the strain of mastication will soon force the abutments out of line.

The final reason for many failures in this work is that a great many dentists who are not skillful enough to construct their own bridge-work send it out to dental laboratories, where their expert manipulators construct the bridge on plaster models. Such work can not be too strongly condemned, and no dentist should attempt bridge-work if he can not make it himself. The more fitting we do in the mouth of our patient, the more satisfactory will be the finished product, working to plaster casts and models is not an acurate means of doing good and reliable work.

In concluding, I have no reserve in saying that there is no form of replacement, that can approach in appearance, comfort and utility that of a well conceived, proportioned and constructed bridge. I may also add with propriety, another accompaniment to a finished bridge, that of cleanliness.

To be a successful bridge-worker, a man must be a thinker, have keen judgment, and discernment, for so many complicated cases arise and conditions differ that each one requires a special study. He must be a good mechanic, a metallurgist, and above all, an *artist*.

DISCUSSION.

Dr. N. S. Hoff: The essayist has opened up several phases of the subject of bridge-work. The artistic aspect of the work is one that would take a long time to consider. I do not feel that he has exhausted that feature of the subject at all, in fact he has only referred to it.

One suggestion comes to my attention, with this aspect in view, in regard to which I would take issue with the essayist. He suggests that in all cases where anterior teeth are to be used for abutments the only admissible form of attachment is that of a Richmond crown, that is a banded crown with a porcelain face. I do not believe that this is the only kind of attachment for bridge-work on these teeth. Such crowns, however artistically the shades and shape of teeth when inserted may have been, will in a few months or years be entirely out of harmony with their surroundings. The color of porcelain may not change, but the adjacent teeth will certainly change, taking on color with age. It is a very easy matter to detect a gold soldered porcelain-faced or Richmond crown in the mouth after being worn a few years, and for this reason Richmond crowns for the anterior part of the mouth are not always the only or best thing. Richmond crowns, built up in porcelain work rather than soldered, are much more desirable artistically. The colors are more easily chosen and a porcelain crown so made is much more impervious to the fluids of the mouth and less likely to discolor, much more artistic results can be thus obtained. I think also, with all deference to the essayist, that an open-face gold crown attachment is often much to be preferred to a Richmond crown, particularly where the tooth is sound. The cutting off of a fully developed and perfect crown to make an attachment for a bridge is one of the things to which I can not often bring myself, I only do it under very great protest. I don't think anybody has a right to do this in every case, but only in special cases. The majority of people for whom we are asked to make bridges, are people who take care of their mouths properly, and for anyone who will take proper care of his mouth the open face crown properly fitted will provide a secure bridge attachment without mutilation or artistic offense. I have made a good many of these open-face crowns as attachments for bridge-work and have had but one failure and that was in the mouth of one who would not use a tooth brush in spite of all my remonstrances. This was no fault of the method or the construction, although it may be true that many of these failures result because the crowns have been imperfectly made and fitted. I feel that the essayist needs to revise his decision in that direction, I don't want to question his ability and skill on this kind of attachment and I know he is not alone in this position, but I feel from practical experience that these open-face crowns are very useful, they have served me so well that I shall continue to use them as a rule rather than cut off perfect crowns. It is not necessary to mutilate the tooth, it is necessary to shape it somewhat of course, but never necessary to remove all the enamel. It is necessary that they be made strong and solid to resist the strain put upon them, but this can be done without interfering with the interdental spaces to the extent of obliteration as the essayist says. These open-face crowns can be so adjusted that they do not destroy the natural appearance of the teeth upon which they are adjusted and they can be made without being objectionably conspicuous. The tooth does not suffer in discoloration as much as if cut off and a porcelain crown put in its place. For these reasons I feel that the open-face crown is at least worthy of consideration. The other attachment of the bridge should be a solid gold crown. The cap is most desirable because it gives stability to the whole bridge, and is because of its position admissible esthetically.

Dr. W. H. Dorrance: I do not sympathize with bridge-work to any large extent, I believe there is a larger percentage of bridge-work put in where it should not be than there is where it should be. The mistake lies in the lack of judgment in regard to the matter of bridge-work. People want it; they say, "I can have a bridge put in here and will not have to wear a plate;" so the dentist will put one in whether he can or not, provided he is paid for it, for that is what he is after. Of course this is not the case with all practitioners and bridges are put on which have been a great help to the wearer. The fact that we are uniting two teeth together and that we are putting upon them pressure in only one direction, where it was intended they were to move in all directions, causes many unfavorable conditions. How often in removing teeth to which bridge-work has been attached is exostosis found on the roots. I claim bridge-work should be put on only as a last resort. It can not be any too well done and whether we use open-face crowns or Richmond crowns and to my mind the Richmond crown is not open to all the objections which Dr. Hoff claims, of course all work will fail and can not last forever, but a perfect piece of work is the thing to be desired.

Development in a tooth goes on much longer I think than Dr. Logan claims. The deposit of secondary dentin is proof of this fact. The destruction of a pulp for attachment of a bridge is not the thing to do. I would refuse to put in a bridge which would involve the destruction of the pulp. I should grind off the enamel of teeth for the attachment of a crown, and should make a crown to fit that tooth, keeping it alive, cementing the crown on with gutta percha, if necessary I should use small lugs and a nut to adapt a crown to the tooth. As soon as you take off the enamel the tooth is doomed, and it will require constant treatment to keep it alive. Where there are only two or three teeth alive and in condition to be used as attachments for bridges, let the patient undertand the situation entirely before making an expensive bridge. Let them understand that it will last only six, eight or ten years. I have seen some bridges which were made under very favorable circumstances taken off at the end of two or three years. I certainly think there are other methods of bridge-work, saddles, etc. Patients should be made to understand the length of time which a bridge is likely to be servicable. I do not see why they should not be suited, but I certainly should discountenance the destruction of a healthy pulp. I remember a case in practice where a lady desired four gold crowns to be placed in the mouth, two of them being in a conspicuous position. She would not have any work done at all unless she could have the crowns. I finally consented to place one gold crown upon the upper first right bicuspid. The tooth might have been saved several years by filling. Dr. Logan has presented a very good paper although I do not agree with him in all things, but I don't think he has gone a great way out of correct practice. A man to be a good worker in crown or bridge-work must have a good head and good hands, and I don't think any man has any business making bridge-work who at least has not the skill required to construct a good rubber plate.

Dr. J. Taft: There is no question that crown and bridge-work has been carried to excess, work has been done where it should not have been done. But why this desire to substitute bridges, etc., instead of other methods of artificial substitutes? How often patients say to us, "I never knew what trouble with my teeth was until I began to wear artificial plates." It is not every dentist who can construct and apply artificial dentures. Then again all patients are not careful in the care ot their plates, it is very necessary that the mouth should receive proper attention. Often in a very little while after a plate is inserted such changes take place in the mouth as to render it almost worthless. It is because of this no doubt that many patients are anxious to have a more desirable thing, they have been told that where crowns and bridges have been inserted they do not require to be taken out and cleaned as in the case of plates. So it is very natural that the people desire something which will give less annoyance than ordinary plates. It is not true that all bridges serve a valuable purpose and that the gums always retain their healthy condition, but trouble is probably no more frequent than from the wearing of plates.

One trouble arising from the wearing of bridges may be caused by throwing more stress upon a tooth to which attachment is made than was intended. Usually the teeth rally and come up to the requirements forced upon them, but occasionally they give away. I have seen many bridges which had been in use for years where there was no irritation whatever.

Another source of trouble is that perhaps they are not so easily cleansed and kept in a proper condition as a plate which can be removed from the mouth and cleansed. Many persons are exceedingly careless in the care of their teeth. I have seen teeth entirely em bedded in the refuse round about them.

In regard to one or two points presented by the paper I think some exceptions should be made. I understood essayist to say it was proper enough sometimes to cut off a sound tooth for the sake of attachment for one end of the bridge. If the essayist entertains that idea I hope he will abandon it in the near future and never do it again. This brings to mind a case where a prominent practitioner cut off three sound teeth for attachments for a bridge. They were cut off just outside the margin of gums and crowns built on them. I would seriously object to that kind of a procedure in my own mouth, and I think it a faulty method, and I hope Dr. Logan will discountenance it as soon as he can and find a better way. That there are better ways I do not question. I have personally found the method devised by Dr. Carmichael, of Milwaukee, a conservative, esthetic and secure method of making attachment to the cuspid teeth without mutilation.

I have no sympathy with the idea that when a tooth is once formed the pulp is of no consequence, if so, nature would have provided for its destruction. Many a time the teeth are worn down beyond the limits of the original pulp chamber and that portion of pulp chamber is filled up by a secondary deposit of dentin. Again, pulp stones is evidence of the fact that the pulp is active after the tooth is erupted. I do think the essayist wrong in that respect. All scientists recognize the fact that the odontoblasts remain on the surface of pulp long after the tooth is formed.

Dr. W. H. Jackson: I am not very enthusiastic over bridge-work. I put them in where I have to, but not where I always want to. There are certain classes of cases in which you can feel more certain of success than in others. Bridges are less liable to be successful in younger than in older people, because the teeth become more nearly ankylosed in older than in younger patients, consequently the teeth have less motion and become less liable to destruction. There is another thing that is not always given the proper amount of consideration, and that is, that teeth which have lost their pulps have a greater tendency to recession of the gum, an elongated appearance of the tooth takes place, exposing the gingival line of the crown. If you put a crown over a tooth with a live pulp the gums do not recede as much. I have put on seven crowns in one lady's mouth. It is now twenty years since I put on the first one. The last one the gum has not receded, the pulp is alive, all the others the gum has receded, the pulps were killed. I put on a crown to-day without killing the pulp. I always avoid killing a pulp if I can, for this reason if for no other.

As to there being no other way for attaching a bridge to the cuspids and incisors than a Richmond crown, I can not agree. I should always have one of the supports a solid capped crown; whatever it be, let it be solid. If a banded or open-faced crown, the band may slip over into the gum.

After getting a capped crown for one abutment I sometimes extend the other end over on to another tooth, or if it be decayed, I put in a gold filling leaving a hollow space on top in which rests the other end of the bridge, or if no cavity, dress out a place on top of the tooth for same. I do this so the two teeth may work separately. I can show bridges which have been in use for as much as twelve years, which are good and solid to-day, and which were put in in that way. The end simply rests into a groove cut into a filling of the tooth and supported by sufficient gold to withstand mastication on that point. I do not believe in mutilating any of the teeth for supports and attachments. I think one good tooth is worth more than all the bridges which could be put in a mouth.

Dr. L. P. Hall: I have not had very much experience myself in bridges. I put one on about eight years ago and it took itself off the other day, but I think perhaps the fault of that was I did not adapt my band and capped crown as well as I ought to have done. I put on another one a few years ago and I guess that is there yet, it was supposed to have been a removable bridge, I had to dig about an hour to get it out from under a huge deposit of salivary calculus the last time I saw the patient. The most experience I have had has been in swinging in centrals and laterals upon good live teeth. I use a heavy band, but cut so as to show as little gold as possible and soldered my dummy to that. In these cases I do not think it is well to cut off the tooth or to devitalize the pulp. If the tooth were devitalized I should put on a Richmond crown. Dr. Hoff said he did not like a Richmond crown made with gold because of the change in color, would it be any more likely to change in color than the facing swung in? The porcelain teeth do not change so much as the surrounding natural teeth do? In a plate of course, when the teeth change in color, it is very easy to make new plates to match the teeth.

Dr. Logan closes the discussion: With all due respect to the gentlemen who have spoken, I still hold my same opinion. Richmond crowns are the most artistic if properly made. They are much more artistic than the open-faced crown. If a Richmond crown is made as it should be the saliva has no chance to penetrate between the backing and the facing. That the porcelain crown is more impervious than the Richmond I do not believe, because the Richmond is absolutely impervious.

Dr. Hoff stated that it was not necessary to dress a tooth very much ordinarily to allow for the open-faced crown. The cuspid is the most bell-shaped crowned tooth in the mouth, and it is absolutely impossible to solder a facing to a band and slip it over the cuspid tooth without grinding off a considerable amount of the enamel.

In reference to the secondary deposit of dentin I would like to know what is the pathological condition that would cause the dentin to form under the conditions stated. I will admit that there is a greater amount deposited where you have some pathological condition. At the same time this deposit is constantly going on. Dr. Dorrance also said that a tooth was liable to die if the enamel was removed. I had seventeen crowns inserted in my mouth while here in college. In every case the enamel was more or less removed. This is six years, I have not yet a dead tooth in my head. Of course all patients are not alike, in some cases where the patient is anemic the pulp will die. If you take the proper measures afterwards to cauterize the teeth with silver nitrate there will be no trouble.

Dr. Taft spoke of work being permanent if kept cleanly, I do not care how thoroughly a bridge or crown is made, if the patient is not careful in the attention given the mouth the best of work will fail. I claim it is not fair to test a bridge under such conditions.

Returning again to this matter of cutting off a cuspid tooth, Dr. Hoff claims it is a mistake, and that it is impossible to thoroughly adapt a backing to a facing.

I claim you can, and do it perfectly. The color may not remain the same but by using a little judgment and allowing for the change of color they will conform with the appearance of teeth later in life as the natural teeth become darker.

Dr. Jackson spoke of inserting a bridge and leaving one end free. This is all right if the patient bites direct. But what will happen if the patient bites to one side? He also said that where the tooth was dead it was much more liable to the recession of the gum tissue. I fail to see where there is a direct connection between the gum and the pulp. The peridental membrane receives its blood supply just the same when the pulp is dead and I think you will find just as much absorption on perfectly sound teeth so far as the pulp is concerned, as upon dead teeth. This condition is very often due to the collection of tartar upon the roots. I would like to ask Dr. Jackson to explain this.

Dr. Jackson explains: The teeth have not that vitality that they have when the pulp is alive. From experience of thirty-five years I have yet to see a case which is contrary to my statement. These are facts. Of course, it may not be always true, neither is it always true where a man does not clean his teeth, that they are lost. I filled two teeth for a man in the month of February, 1866, and I don't think he ever had a brush in his mouth, and when I last saw those fillings they were in perfect condition. There was some condition there which made the teeth immune to decay. So in the case of the recession of the gums which makes a seeming elongation of the tooth, there is some condition taking place in the pericemental tissues after the pulp is destroyed which causes this peculiarity.

